# Draft Genome Sequence of “*Acidibacillus ferrooxidans*” ITV01, a Novel Acidophilic Firmicute Isolated from a Chalcopyrite Mine Drainage Site in Brazil

**DOI:** 10.1128/genomeA.01748-15

**Published:** 2016-03-17

**Authors:** Hivana Dall’Agnol, Ivan Ñancucheo, D. Barrie Johnson, Renato Oliveira, Laura Leite, Victor S. Pylro, Roseanne Holanda, Barry Grail, Nelson Carvalho, Gisele Lopes Nunes, George Tzotzos, Gabriel Rocha Fernandes, Julliane Dutra, Sara Cuadros Orellana, Guilherme Oliveira

**Affiliations:** a Vale Institute of Technology, Belém, Pará, Brazil; b College of Natural Sciences, Bangor University, Bangor, United Kingdom; c René Rachou Research Center, Fiocruz, Belo Horizonte, Minas Gerais, Brazil

## Abstract

Here, we report the draft genome sequence of “*Acidibacillus ferrooxidans*” strain ITV01, a ferrous iron- and sulfide-mineral-oxidizing, obligate heterotrophic, and acidophilic bacterium affiliated with the phylum *Firmicutes*. Strain ITV01 was isolated from neutral drainage from a low-grade chalcopyrite from a mine in northern Brazil.

## GENOME ANNOUNCEMENT

Extreme acidophiles include a large number of species of bacteria, archaea, and some eukaryotic microorganisms that have an optimal pH for growth of <3.0. They are commonly found in geothermal and volcanic areas and also in metal mining sites (mineral spoils and drainage waters [1]). Acidophilic bacteria include species of sulfide mineral-oxidizing Gram-positive bacteria, including low-G+C-content *Firmicutes* of the genera *Sulfobacillus* and *Alicyclobacillus*. These organisms represent a storehouse of physiological, metabolic, and genetic diversity, with many species being able to switch between autotrophic and heterotrophic metabolism or to grow mixotrophically. Many species can use ferrous iron, elemental sulfur, and reduced inorganic sulfur compounds as electron donors (lithotrophy), and some species can also obtain energy from organic substances (organotrophy) (1). Some apparently novel strains of acidophilic iron-oxidizing *Firmicutes* were previously isolated from sulfidic mineral waste (2), and more recently, the novel genus “*Acidibacillus*” and binomial species “*Acidibacillus ferrooxidans*” proposed for these and other closely related isolated mesophilic species that oxidized ferrous iron but not elemental sulfur (3). One of these novel strains, ITV01, had been isolated from an acidic stream draining from a low-grade chalcopyrite ore pile in a mine located in the southern border of the Carajás belt, Pará State, Brazil, in December 2013.

Sequencing of the “*A. ferrooxidans*” ITV01 genome was performed on an Ion Personal Genome Machine (PGM) (Thermo Fisher Scientific). Two libraries were produced. The first was constructed form DNA fragmented by sonication and the second from enzymatically fragmented genomic DNA. The two libraries generated 2,342,447 and 2,502,004 reads, respectively. A total of 1.7 Gb of fragment reads were produced, with an average length of 300 bp. Raw sequences were trimmed based on quality criteria (Phred *Q* ≥ 20) using PRINSEQ, and homopolymers >6 bp were discarded (4). After trimming, 88% of the reads of the first library and 90% of the second library passed quality control (~567-fold genome coverage). Draft *de novo* genome assemblies were generated using Newbler (454 Sequencing) and SPAdes (5), merged using the Mix Pipeline (6). The final assembly produced 72 contigs, with a total length of 3,164,591 bp and a contig *N*_50_ of 104,067 bp. The average DNA G+C content was 52.1%.

Protein-coding sequences (CDSs) and tRNA genes were predicted by PATRIC (7). Functional annotations of 3,402 protein-coding genes were obtained, with 1,786 genes having known functions and 1,616 encoding hypothetical proteins. In addition, one copy each of 16S rRNA and 23S rRNA, two copies of 5S rRNA, and 58 tRNA genes were predicted using RNAmmer (8) and tRNAscan-SE (9).

### Nucleotide sequence accession numbers.

This whole-genome shotgun project has been deposited at DDBJ/EMBL/GenBank under the accession no. LPVJ00000000. The version described in this paper is version LPVJ01000000.
